# Single-Port Robotic-Assisted Excision of the Urachal Remnant in an Adult Female: A Case Report

**DOI:** 10.7759/cureus.53235

**Published:** 2024-01-30

**Authors:** Kevin D Kunitsky, Mustafa Almajedi, Elizabeth Snajdar, Parker Adams, Ryan Nelson

**Affiliations:** 1 Department of Urology, Kansas City University, Kansas City, USA; 2 Department of Urology, McLaren Macomb, Mount Clemens, USA; 3 Department of Urology, Henry Ford Macomb, Clinton Township, USA

**Keywords:** intravesical urachal cyst, urachal anomalies, surgical robotics, single-port laparoscopic surgery, urachal remnant

## Abstract

Urachal anomalies and their associated disease processes are quite rare in pediatric populations and even rarer in adults. Although often asymptomatic, patients with symptoms can be treated with a combination of surveillance, antibiotics, and sometimes surgical resection. In this case, we describe our experience using the single-port robotic approach for the excision of a symptomatic urachal remnant. The patient presented with a chief complaint of urinary frequency, dysuria, intermittent hematuria, and right flank pain. A CT scan of the abdomen and pelvis revealed a bladder wall thickening at the dome of the bladder measuring 2.6 x 3.6 x 1.5 cm with concerns for adenocarcinoma. The patient subsequently underwent a biopsy, which was benign. The patient's symptoms persisted, and she elected to undergo surgical resection. Postoperatively, her symptoms resolved, and she was satisfied with her treatment outcome. This case exemplifies the feasibility of the single-port robotic approach to urachal remnant excision, with further applicability to simple transabdominal robotic bladder surgery.

## Introduction

Urachal anomalies are rare, occurring in only 0.063% of adults, resulting from a failure of obliteration during embryological development [1-3]. Normally, by the 12th week of development, the urachus obliterates and becomes the median umbilical ligament. This process occurs through the extension of the proximal allantois into the urogenital sinus, with the remaining sinus encircled by the umbilical cord. The allantois then emerges from the cord to form the urachus, a connection between the umbilicus and the apex of the bladder.

Many different urachal anomalies can result from failed obliteration. The discovery of urachal remnants in adults has increased with improvements in imaging modalities such as computed tomography (CT) or magnetic resonance imaging (MRI). Surgical management is sometimes the treatment of choice if the remnant is symptomatic or if there is concern that the remnant is associated with carcinoma, which is believed to be due to mucosal exposure to recurrent infection, inflammation, and urinary stasis [1,4,5].

Clinical manifestations are dependent on the type of urachal anomaly and are increasingly rare in adults. In this case, we describe our findings in an adult patient who developed recurrent urinary tract infections as well as hematuria. She was diagnosed with a urachal remnant, which was treated with single-port robotic surgical resection. Although previous cases of urachal anomalies have been reported in adults, there is still limited literature regarding this condition.

## Case presentation

A 34-year-old female with a past surgical history of appendectomy and a single cesarean delivery eight years prior and no relevant past medical history or family history presented to the emergency department with sharp progressively worsening right flank pain for one day. She reported associated gross hematuria, urinary frequency, and dysuria, in addition to nausea. Physical examination demonstrated suprapubic and right flank tenderness.

**Figure 1:**
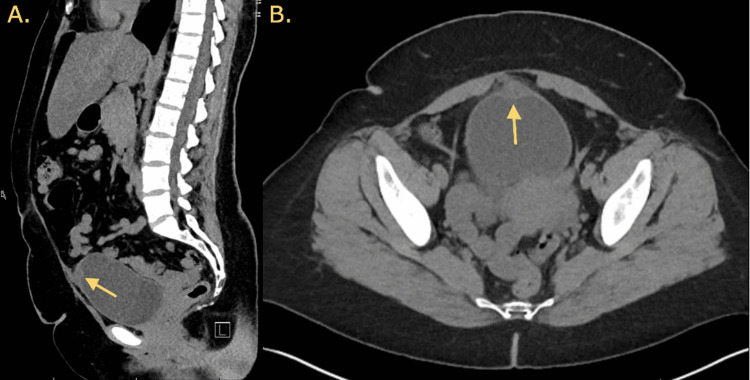
Findings of the CT of the abdomen and pelvis. A. Sagittal view of the CT of the abdomen and pelvis. B. Axial view of the CT of the abdomen and pelvis.

CT imaging of the abdomen and pelvis without contrast demonstrated findings suspicious for possible urachal remnant adenocarcinoma at the bladder fundus (Figure 1). Lab workup revealed urinalysis positive for 4 RBCs/hpf as well as rare bacteria; comprehensive metabolic panel and complete blood count were both unremarkable. Urology was consulted for further evaluation.

Further questioning revealed a history of two urinary tract infections in the prior three months and intermittent gross hematuria. The urine culture was negative and the patient was advised to follow up as an outpatient for cystoscopic evaluation and discussion of possible robotic resection based on cystoscopy findings. A cystoscopic evaluation revealed a soft tissue urachal remnant at the dome of the bladder. Interventional radiology consultation was arranged for an ultrasound-guided urachal remnant biopsy. A needle core biopsy was taken with pathology, notable for scant fibrous tissue only.

Due to continued symptoms, the patient elected to proceed with robotic urachal remnant excision. After anesthesia was induced, the patient was placed in the supine position and the genitals as well as the abdomen were prepped and draped in a sterile fashion. A Foley catheter was placed and a 5 cm horizontal midline incision was made just above the umbilicus. Dissection was carried down to the fascia, which was incised horizontally. The rectus abdominis muscle was split, the peritoneum was identified and entered, and the single robotic ring was placed into the intraperitoneal cavity. The globe was then connected, the patient was insufflated, and the robot was then docked in standard fashion. The procedure was completed as a true single port, and no additional ports were needed.

**Figure 2:**
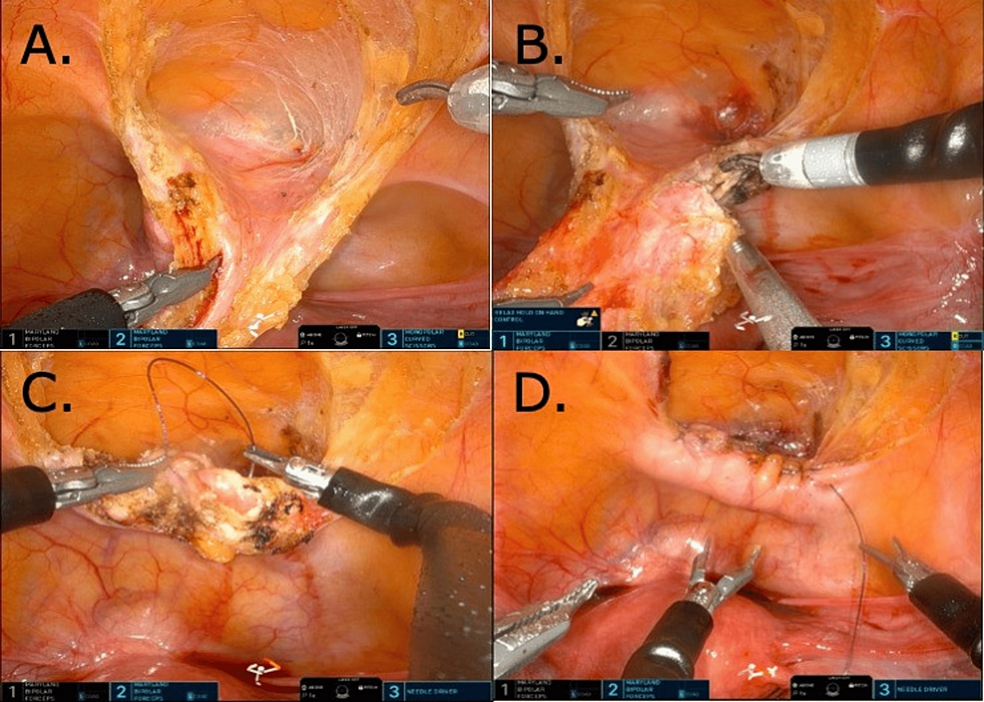
Intraoperative visualization of urachal remnant. (A & B) Mobilization and resection of urachal remnant. (C) Suturing of the cystotomy. (D) Completed repair of the cystotomy.

The median umbilical ligament was identified and incised cephalad, following this dissection plane down to the dome of the bladder where a cystic-like structure was identified (Figure 2). The remnant was excised along with the involved part of the bladder completing the partial cystectomy. The specimen was retrieved and sent for analysis. Hemostasis was achieved using electrocautery.

Using a 6-inch 3-0 V-Loc, the bladder was closed in a running fashion ensuring mucosal apposition. Next, an imbricating second layer closure was completed with another 6-inch 3-0 V-Loc. The bladder was then leak tested with approximately 180 cc of saline and no leak was identified; anastomosis was watertight.

After all sutures and instruments were removed, the robot was then undocked and the globe was removed. The fascia was closed in a running fashion with 0-PDS (polydioxanone) suture. The subcuticular tissues were approximated with 3-0 Vicryl, the skin was closed with 4-0 Vicryl in a running fashion. Dermabond was placed on top of the skin, and an abdominal binder was placed on the patient.

The patient tolerated the procedure well without any complications and had an estimated blood loss of 10 cc. The operative time was two hours and 15 minutes. Once stable, the patient was discharged home the same day with a 16 French Foley catheter in place.

Her Foley catheter was removed in the clinic two weeks after the procedure. Pathology findings revealed a urachal remnant and was negative for carcinoma. The patient reported resolution of symptoms at her one-month follow-up post-procedure.

## Discussion

The first case of a urachal remnant was described as early as 1550 [5]. In normal embryological development, the urachus is a normal remnant of the bladder dome. Many different urachal anomalies can occur due to failed obliteration with some examples including a patent urachus, a urachal sinus, a urachal diverticulum, and a urachal cyst. Acquired diseases relating to these anomalies include infection as well as neoplasm.

Diagnosis of urachal remnant anomalies and their associated manifestations can be difficult due to no distinct symptomatology; however, diagnosis is increasing with improvements in various imaging modalities [1]. Patients may present with various urinary symptoms, such as hematuria, dysuria, retention, and suprapubic pain, whereas laboratory evaluation, including urinalysis and culture, is negative in up to 80% of cases [6,7]. Due to these more vague symptoms, other diseases are often suspected first as the likely cause [8]. In this case, our patient presented with urinary frequency, hematuria, dysuria, and right flank pain.

Ultrasound and CT are often the imaging modalities of choice to identify possible remnants; however, both can fail to differentiate between inflammatory and neoplastic processes. Abscesses can mimic the appearance of neoplasms and vice-versa, thus both possibilities need to be included in the differential diagnosis. Other possible differentials include hernia, hematoma, appendicitis, and peritonitis [8]. CT findings in our case were suspicious for possible urachal remnant adenocarcinoma at the bladder fundus. Once a negative biopsy was obtained, robotic excision was pursued for symptomatic management.

Management in adult patients typically includes antibiotic therapy to treat possible infections, followed by primary excision or secondary excision if abscess drainage is first required. Previous work in children has shown that up to a third of patients with infected cysts who underwent resection developed recurrence [9]. Other studies have published data that demonstrated that 25% of cystic urachal lesions in adults are malignant with 20% of those cases presenting with metastatic disease, including those that were asymptomatic [2]. This was suggested to be due to the gradual malignant transformation of a urachal remnant, and that risk continually increases with age [2,10,11]. Nitti et al. thus advocate for surgical excision of urachal anomalies if they persist into adulthood, regardless of symptomatology [1].

Various approaches can be used for excision with transverse or midline infra-umbilical incisions. Pure laparoscopic and robotic-assisted approaches have also been previously described. In this case, our patient underwent a single-port robotic-assisted resection of her urachal remnant. Advantages of utilizing this modality compared to the previously mentioned approaches include improved ergonomics, regionalized surgery, supine positioning, decreased docking time, and a single incision while disadvantages include restricted freedom of movement due to a fixed entry point. The advantages have the potential to decrease operative time and postoperative pain, as well as improve cosmesis, which was particularly important to our patient in this case.

## Conclusions

Urachal remnants are rare lesions often diagnosed in pediatric patients. In this case, the patient was an adult female who presented with urinary frequency, dysuria, hematuria, and flank pain. She was managed with surgical resection using a single-port robotic-assisted approach, which resolved her symptoms and the patient was satisfied with treatment results at the one-month follow-up period. The single-port robot is a feasible approach to urachal remnant excisions with the potential of decreased operative time and postoperative pain with improved cosmetics. This approach could also be generalized to other simple transabdominal robotic bladder surgery. Further studies will be needed to show the reproducibility of this approach and gain a better understanding of the potential benefits or limitations compared to a multiport approach.

## References

[1] Nitti M, Martin AD, Reifsnyder JE, Ortenberg J, Westerman ME, Roth CC (2022). The urachus: current concepts. AUA Update Series.

[2] Ashley RA, Inman BA, Routh JC, Rohlinger AL, Husmann DA, Kramer SA (2007). Urachal anomalies: a longitudinal study of urachal remnants in children and adults. J Urol.

[3] Siow SL, Mahendran HA, Hardin M (2015). Laparoscopic management of symptomatic urachal remnants in adulthood. Asian J Surg.

[4] Naiditch JA, Radhakrishnan J, Chin AC (2013). Current diagnosis and management of urachal remnants. J Pediatr Surg.

[5] Mahato NK, Mittal MM, Aggarwal R, Munjal KM (2010). Encysted urachal abscess associated with a premalignant lesion in an adult male. Urotoday Int J.

[6] Blichert-Toft M, Nielsen OV (1971). Congenital patient urachus and acquired variants. Diagnosis and treatment. Review of the literature and report of five cases. Acta Chir Scand.

[7] MacNeily AE, Koleilat N, Kiruluta HG, Homsy YL (1992). Urachal abscesses: protean manifestations, their recognition, and management. Urology.

[8] Tazi F, Ahsaini M, Khalouk A, Mellas S, Stuurman-Wieringa RE, Elfassi MJ, Farih MH (2012). Abscess of urachal remnants presenting with acute abdomen: a case series. J Med Case Rep.

[9] Agatstein EH, Stabile BE (1984). Peritonitis due to intraperitoneal perforation of infected urachal cysts. Arch Surg.

[10] Tanguturi Yella V, Tanguturi Yella SS, Kota KS, Tanguturi Yella SH, Thangaraju P (2023). A very rare disease of patent urachus cyst with vesico-umbilical urinary fistula in adults: a case report and short review. Cureus.

[11] Ryan PC, Kelly C, Afridi I (2023). Surgical treatment of urachal remnants in an adult population—a single-centre experience. Ir J Med Sci.

